# A three-dimensional co-culture system to investigate macrophage-dependent tumor cell invasion

**DOI:** 10.14440/jbm.2016.132

**Published:** 2016-07-24

**Authors:** Amy R. Dwyer, Lesley G. Ellies, Andrea L. Holme, Fiona J. Pixley

**Affiliations:** ^1^School of Medicine and Pharmacology, University of Western Australia, Perth Australia; ^2^School of Anatomy, Physiology and Human Biology, University of Western Australia, Perth, Australia; ^3^Centre for Microscopy, Characterisation and Analysis, University of Western Australia, Perth, Australia

**Keywords:** macrophage, motility, 3D co-culture, invasion, mammosphere

## Abstract

Macrophages infiltrate cancers and promote progression to invasion and metastasis. To directly examine tumor-associated macrophages (TAMs) and tumor cells interacting and co-migrating in a three-dimensional (3D) environment, we have developed a co-culture model that uses a PyVmT mouse mammary tumor-derived cell line and mouse bone marrow-derived macrophages (BMM). The Py8119 cell line was cloned from a spontaneous mammary tumor in a Tg(MMTV:LTR-PyVmT) C57Bl/6 mouse and these cells form 3-dimensional (3D) spheroids under conditions of low adhesion. Co-cultured BMM infiltrate the Py8119 mammospheres and embedding of the infiltrated mammospheres in Matrigel leads to subsequent invasion of both cell types into the surrounding matrix. This physiologically relevant co-culture model enables examination of two critical steps in the promotion of invasion and metastasis by BMM: 1) macrophage infiltration into the mammosphere and, 2) subsequent invasion of macrophages and tumor cells into the matrix. Our methodology allows for quantification of BMM infiltration rates into Py8119 mammospheres and demonstrates that subsequent tumor cell invasion is dependent upon the presence of infiltrated macrophages. This method is also effective for screening macrophage motility inhibitors. Thus, we have developed a robust 3D *in vitro* co-culture assay that demonstrates a central role for macrophage motility in the promotion of tumor cell invasion.

## BACKGROUND

While 2D cell culture monolayers facilitate examination of intracellular signaling cascades and cell behavior, this approach largely ignores the more complex structural organization present in the normal physiological setting. Cells behave differently in a 3D environment, in part because the extracellular matrix is a key regulator of normal homeostasis and tissue phenotype [1] . It has long been known that breast epithelial cell lines form highly organized, growth arrested acinar structures when grown in conditions of low adhesion such as within the basement membrane matrix, Matrigel. These mammary organoids are analogous to the ductal architecture of normal breast tissue [2]. Examination of cellular behavior and signaling using this 3D cell culture approach improves the relevance of cell-based assays [3, 4]. Non-cancerous human breast epithelial cell lines, such as MCF10A, can be induced to become invasive upon expression of oncogenes [5]. Similarly, co-culture of mammospheres with other cell types such as macrophages can also induce invasion [6]. As tumor associated macrophages (TAMs) are key players in the regulation of breast cancer metastasis [7, 8], the generation of mammospheres derived from breast cancer cells and subsequent infiltration of the spheroids by macrophages is an attractive model to examine mechanisms by which TAMs assist in tumor progression to invasion and metastasis.

Macrophages are fully differentiated cells of the mononuclear phagocytic lineage and they have coordinated functions in development, tissue remodeling and homeostasis. Due to their integrated function with parenchymal cells in tissues, macrophages contribute to the pathogenesis of a number of chronic diseases [9]. In breast cancer, TAMs have been shown to promote tumor invasion and metastasis by establishing a paracrine chemotactic loop whereby breast carcinoma cells secrete colony stimulating factor-1 (CSF-1) and TAMs secrete epidermal growth factor (EGF) to induce co-migration of both cell types [10, 11]. CSF-1 receptor (CSF-1R) signaling is currently under investigation as a therapeutic target [12, 13] and we have identified CSF-1-stimulated macrophage motility, through PI3K p110δ and SFKs, as a promising specific target to prevent tumor invasion [14-16].

**Figure 1:**
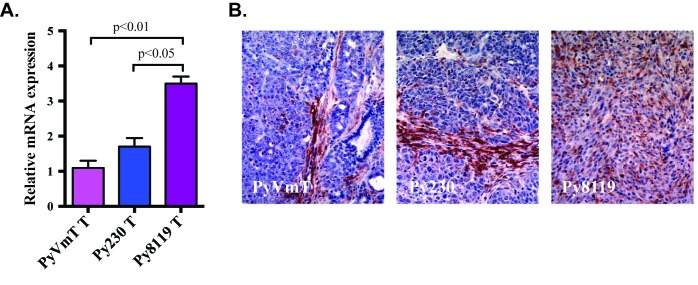
**CSF-1 expression by PyVmT cell line derived tumors**. CSF-1 expression was measured by microarray. A. Spontaneous PyVmT tumors - PyVmT T; Py230 well differentiated luminal tumors - Py230 T; and Py8119 aggressive EMT tumors - Py8119 T. Data are means ± SEM from 6 tumors per group and were analyzed by one-way ANOVA and Holm-Sidak’s multiple comparisons test. **B**. Representative sections of PyVmT spontaneous tumor and Py230 and Py8119 cell line-derived tumors stained with anti-F4/80, a macrophage marker (brown), and counterstained with hematoxylin (blue).

Transwell or collagen overlay assays are standard methods used to measure macrophage and tumor cell co-migration [10, 15]. A major advantage of using mammary spheroid assays over the transwell or collagen overlay assays, is that the tumor cells are organized into a 3D structure, which mimics the pathophysiological development of tumors *in vivo.* In addition, co-culture of macrophages with the mammospheres allows dissection of tumor cell-macrophage interactions in the promotion of tumor invasion by macrophages [6]. Despite these advantages, this method has been underexploited. Furthermore, the few reported assays have either co-cultured human breast cancer cell lines with mouse macrophages [17-19] or used human monocyte-derived macrophages with a human breast cancer cell line [6]. Co-culture of mouse BMM with a mouse mammary tumor-derivettd cell line is both species-specific and more experimentally tractable. Thus, we have developed a mouse mammary tumor cell-mouse BMM co-culture model to examine the role of BMM motility in encouraging tumor cell invasion in a more physiologically relevant context. We have chosen the Py8119 mammary tumor cell line isolated from a spontaneous tumor in a Tg(MMTV:LTR-PyVmT) C57Bl/6 mouse [20] because it represents aggressive breast cancer cells that have undergone epithelial to mesenchymal transition. Quantitative PCR revealed high levels of CSF-1 message in these cells compared with more differentiated luminal breast cancer cells (**Fig. 1A**). Furthermore, the CSF-1 message levels are consistent with the large numbers of macrophages recruited *in vivo* (**Fig. 1B**). Our *in vitro* model allows dissection of two critical steps involved in the promotion of tumor cell invasion and metastasis by BMM; 1) macrophage infiltration into the Py8119 mammosphere, and 2) subsequent invasion of co-migrating BMM and tumor cells into the surrounding matrix.

The protocol described herein outlines the necessary steps 1) to routinely generate Py8119 mammospheres of reproducible size and shape, 2) to produce consistent levels of CellTracker Red-labeled BMM infiltration into the mammospheres, and 3) to reliably induce invasion of tumor cells with their accompanying macrophages into the surrounding matrix. Here we show that tumor cell invasion is dependent on the presence of infiltrated macrophages. We have recently applied this methodology to examine the role of macrophage motility in tumor invasion and have shown that the degree of macrophage infiltration into Py8119 mammospheres, and the degree of invasion by breast cancer cells and macrophages into the matrix is directly proportional to their speed of migration (manuscript in preparation).

While we have examined the role of macrophages in a breast cancer model, tumor-associated macrophages have been shown to promote tumor progression in a wide range of solid tumors [21]. Thus, this organoid/BMM co-culture system could be extended to other 3D tumor models such as pancreatic, prostate and stomach cancer cell line-derived organoids [22-26].

## MATERIALS

### Cells

• Py8119 cells isolated from a spontaneous tumor in a Tg(MMTV:LTR-PyVmT) C57Bl/6 mouse [20]
• Stably transfected EGFP-Py8119 cells (used for live cell imaging and flow cytometry)
• Mouse bone marrow derived macrophages, isolated from tibiae and femurs of C57Bl/6 mice as described previously [16]

### Reagents

• 6-well ultra-low attachment cell culture dish (Corning, cat. # 3471)
• 10 cm Petri cell culture dish (Falcon, cat. # FAL351058)
• 10 cm Tissue culture treated cell culture dish (Eppendorf, cat. # 0030702115)
• 24-well ultra-low attachment cell culture dish (Corning, cat. # 3583)
• 24-well glass bottom dish (MatTek, cat. # P24G-1.5-10-F)
• 35 mm Glass bottom dish (MatTek, cat. # P35G-1.5-14-C)
• Alexa-488-phalloidin (Life Technologies, cat. # A12379)
• ART tips, low retention, 200 µl (Thermo Fisher, cat. # 2069-05-ART 200P)
• B-mercaptoethanol (Sigma Aldrich, cat. # M6250-100 ml)
• Bisbenzimide (Hoechst) 33342 (Sigma Aldrich, cat. # B 2261)
• Bovine serum albumin (BSA; Sigma Aldrich, cat. # A7906-100G)
• Cell culture medium (a-MEM; Life Technologies, cat. # 11900-024)
• Cell recovery solution (Corning, cat. # 354253)
• CellTracker Red CMPTX (Life Technologies, cat. # C34552)
• CSF-1; 120 ng/ml (kind gift of E.R. Stanley)
• Dulbecco’s phosphate buffered saline, 1x (Life Technologies, cat. # 21600-010)
• EDTA (0.5 M) (Amresco, USA, cat. # 0322-500G)
• FACS tubes, 5 ml round bottom, 12x 75 mm (Falcon, cat. # FAL352002)
• Fetal calf serum (FCS; Bovogen, cat. # SFBS-F)
• Filter tips, 1000 µl (Thermo Fisher, cat. # QSPTF112-1000)
• Fungizone (Life Technologies, cat. # 15290018)
• Gentamicin (Life Technologies, cat. # 15750078)
• Glass coverslips (ProSciTech, cat. # G422)
• Glutamax (Life Technologies, cat. # 35050-061)
• Glycine (Merck Millipore, cat. # 1042010250)
• Goat serum (Life Technologies, cat. # 16210-064)
• GS-1101 (Kind gift of Gilead Sciences)
• GW2580 (Calbiochem, cat. # 344041)
• Ham’s F12K Nutrient Mix (Life Technologies, cat. # 21127022)
• L-Asparagine (Sigma Aldrich, cat. # A4159-25G)
• Matrigel Basement Membrane Mix, high concentration (HC), IdEV-Free (Falcon, cat. # FAL354248)
• MITO+™ serum extender (Corning, cat. # 355006)
• Paraformaldehyde (32% PFA) (Electron microscopy services, cat. # 15714)
• Penicillin/Streptomycin (Sigma Aldrich, cat. # P4333)
• Prolong Diamond mounting agent with Dapi (Life Technologies, cat. # P36962)
• Sodium hydrogen carbonate, NaHCO_3_ (Fluke, cat. # 71628)
• Triton X-100 (Sigma, cat. # T8787-50 ml)
• Trizma base (Sigma Aldrich, cat. # T1503)
• Trypan blue (BioRad, cat. # 145-0013)
• Trypsin (0.25%)/EDTA (2 mM) (Sigma Aldrich, cat. # T 4049)
• Tubes, 15 ml, sterile (Thermo Scientific, cat. # 339650)

### Recipes

• *BMM cell culture medium (BMM medium):* Add FCS to 10% of volume of supplemented α-mem (α-mem, 0.01% (v/v) Penicillin/Streptomycin, 4.4 g NaHCO_3_, 0.005% (v/v) Glutamax, 40 mg asparagine, 6.8 µl β-mercaptoethanol, pH 7.2) and 120 ng/ml CSF-1. Store at 4ºC for 4–6 weeks.
• *Py8119 cell culture medium (Growth medium):* To F-12K nutrient medium add 5% FCS, 50 µg/ml gentamicin, 2.5 µg/ml fungizone and 0.1% (v/v) MITO serum extender, and store at 4ºC for 413970591406 weeks.
• *2mM EDTA:* Add 200 µl EDTA to 50 ml sterile 1x PBS, filter through 0.2 µm filter syringe and store at 4ºC.
• *Working Matrigel solution:* Dilute ultra high concentration Matrigel to 8 mg/ml in ice-cold 1 × PBS. Aliquot into 1 ml aliquots and store at −20ºC. The solution must be thawed on ice for at least 6 h prior to use and kept on ice while pipetting since it gels rapidly.
• *Fix buffer:* Add PFA to a final concentration of 2% in 1 × PBS. Make fix solution fresh for each experiment.
• *0.25% Triton X-100:* Dilute 10% Triton X-100 in 1 × PBS.
• *0.1M glycine:* Dissolve 37.5 mg glycine in 5 ml 1 × PBS.
• *Blocking solution:* Prepare a solution containing 1% BSA and 10% goat serum to 1 × TBS, adjust pH to 8.0 and store at 4ºC. For each experiment, add 1:20 Alexa488-phalloidin to the required volume of blocking solution.
• *Tris-buffered saline:* To 800 ml water ad 87.7 g NaCl, 12.1 g Trizma base and adjust to pH 7.4
• *CellTracker Red CMPTX working solution:* Prepare a 10 µM solution in serum-free media.
• *FACS buffer:* Add FCS to a final concentration of 1% FCS in 2 mM EDTA in PBS.

### Equipment

• Cell culture incubator (humidified, 5% CO_2_)
• Biological hood with laminar flow (BH2000 series)
• Cell counter (BioRad, cat. # TC10)
• Cell counter chamber slides, dual chamber (BioRad, cat. # 145-0011)
• Nikon Eclipse inverted phase microscope (Nikon, cat. # TS-100)
• Eppendorf centrifuge with swing-bucket rotor, refrigerated (Eppendorf, cat. # 5804R and cat. # A-4-44 rotor)
• A1Si Confocal microscope (Nikon)
• BD LSR Fortessa TM SORP 5 lasers (355, 405, 488 (100 m Watt), 561, 640 mm), filter set

## PROCEDURE

**Figure 2:**
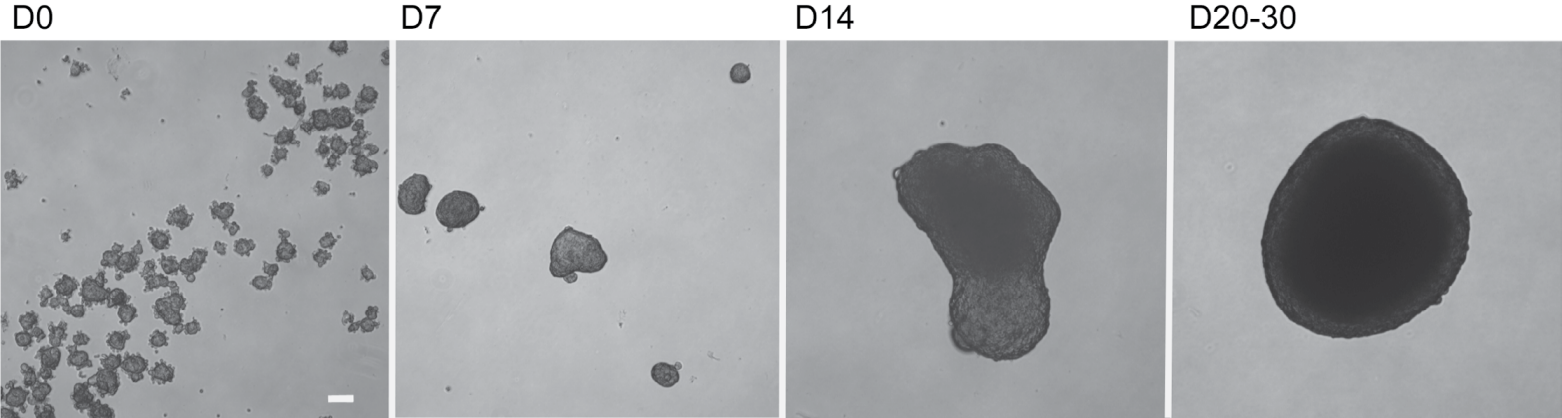
**Py8119 mammosphere formation**. Py8119 cells were plated at 2.5 × 10^4^ cells/ml in 6-well ultra-low attachment plates and grown for 20–30 d before infiltration with BMM. Developing mammospheres were fed with supplemented Ham’s F12K medium every 7 d. Scale bar = 100 µm.

1. Growing Py8119 cells in 2D
  1.1. Grow Py8119 cells on 10 cm tissue culture plates in supplemented F-12K Medium (growth medium).
  1.2. Replace growth medium every 1–2 d depending on cell density. Subculture when cells reach 80139705914090% confluency.
2. Establishing Py8119 mammosphere cultures
  2.1. Wash Py8119 cells with warm PBS and trypsinize with warm Trypsin/EDTA solution for 3 min at 37°C. Neutralize with 5 ml cold growth media and count. Pellet by centrifugation at 800 rpm for 5 min.
  2.2. Determine the volume required for 2.5 × 10^4^ cells/ml and dilute in growth medium.
  2.3. Pipette the desired number of cells into each well of a 6-well ultra-low attachment plate containing 5 ml growth medium per well.
  2.4. Return the plate to the incubator to allow the cells to adhere/aggregate.
  2.5. Mammospheres take 25139705914030 d to grow to approximately 500 µm in diameter (**Fig. 2**)
  2.6. When mammospheres are ready to infiltrate with BMM, gently transfer them in a wide bore tip to a 24-well ultra-low attachment dish with one spheroid per well containing 1 ml growth medium to ensure each mammosphere is exposed to equal numbers of BMM.
**TIPS:** (i) Top up the growth medium every 313970591404 d. (ii) Manually swirl the plate gently, in a circular motion, 213970591403 times per week to encourage formation of 113970591402 mammospheres per well.
**CAUTION:** Py8119 mammospheres are very fragile and can be disrupted by pipetting. Try to minimize handling and, when necessary, use a wide bore tip.
3. Co-culture with BMM
  3.1. Culture mature, adherent BMM (3 d post-extraction) in 10 cm Petri dishes in BMM medium as previously described for 7139705914010 d prior to infiltration [16].
  3.2. Rinse BMM once with warm PBS then stain with 4 ml of 10 µM Cell Tracker Red in serum-free BMM medium for 30 min at 37ºC.
  3.3. Rinse again with warm PBS and allow to recover in complete BMM medium for at least 1 h prior to infiltration.
  3.4. Wash cells with warm PBS and incubate with 4 ml 2 mM EDTA for 20 min at 37ºC, then scrape gently to lift remaining cells from the dish.
  3.5. Count BMM number and dispense 10^5^ cells onto each mammosphere in 100 µl BMM medium.
  3.6. Return dish to the incubator (37 ºC, 5% CO_2_) to allow the macrophages to infiltrate for 3 d.
**NOTES:** After infiltration, mammospheres can be examined by immuno-fluorescence (step 4) or embedded in Matrigel to observe 3D invasion of the ECM (step 5).
4. Immunofluorescent staining of BMM-infiltrated Py8119 mammospheres
  4.1. Gently wash BMM-infiltrated mammospheres with 500 µl warm PBS.
  4.2. Fix with 2% PFA for 20 min at room temperature.
  4.3. Permeabilize with 0.25% Triton X-100 for 10 min at 4°C.
  4.4. Wash 113970591402 times with 0.1 M glycine for 10 min per wash to remove free aldehydes.
  4.5. Block for 60 min in 50 µl 10% goat serum, 1% BSA in 1 × TBS.
  4.6. To examine the actin cytoskeleton, add 2.5 µl Alexa-488-conjugated phalloidin to the blocking solution.
  4.7. Wash 113970591402 times with 1% BSA in 1 × TBS for 5 min.
  4.8. Transfer to a MatTek glass bottom dish.
  4.9. Mount (*e.g.* Prolong Diamond with DAPI) and seal with a glass coverslip.
  4.10. Take representative images of BMM-infiltrated mammosphere on a fluorescent microscope, *e.g.,* Nikon A1Si Confocal Microscope (Nikon PlanApo 10x objective, numerical aperture 0.45, with differential interference contrast (DIC) and 561 nm laser).
  4.11. To quantify macrophage infiltration into the spheroid, determine the area of infiltrating macrophages by thresholding the Cell Tracker Red signal in IMAGEJ software, and express as a ratio of total spheroid area. Correct for BMM number infiltration (%) by loading controls of BMM (6-well dish, not stained), using the IMAGEJ cell counter plugin.
**TIPS:** Use at least three mammospheres per condition for analysis of infiltration.
**NOTES:** (i) Fixed mammospheres may be stored at 4°C for up to 7 d. (ii) Mount cultures no more than 1 d before imaging to prevent accumulation of bubbles in the mounting medium.
5. 3D invasion assay of BMM-infiltrated GFP-Py8119 mammospheres
  5.1. Wash BMM-infiltrated mammospheres in PBS and leave on ice while preparing Matrigel coated plate.
  5.2. Place 24-well glass bottom plate on ice and coat each well with 50 µl of Matrigel (> 8 mg/ml).
  5.3. Transfer plate to incubator and allow the Matrigel to polymerise for 10139705914015 min.
  5.4. Remove the PBS from the mammospheres and gently resuspend in 100 µl of Matrigel and transfer immediately to Matrigel-coated plate.
  5.5. Return the plate to 37°C for 15 min to polymerise Matrigel, then add 1 ml BMM medium to each well.
  5.6. Maintain embedded mammospheres at 37°C, 5% CO_2_ for 7 d before quantification of cell invasion into the Matrigel.
  5.7. Obtain brightfield images (*e.g.,* Nikon Eclipse TS-100 microscope) and then using IMAGEJ, calculate the maximal distance covered by invasive cells by measuring the difference in mammosphere area between d 0 and d 7.
  5.8. Live cell imaging can be carried out in a humidified Tokai Hit stage top incubator (37°C, 5% CO_2_) for prolonged periods of up to 3 d. This allows direct observation of motile BMM as they dig tunnels through the matrix and interact with tumor cells to encourage their motility (**Fig. S1**).
  5.9. For FACS analysis of invading cells, carefully remove media and replace with 1 ml cell recovery solution for 90 min at 4°C.
  5.10. Once Matrigel has liquefied, remove only the remaining spheroid mass with a P200 pipette, ensuring no extra solution is removed.
  5.11. Collect cell recovery solution (minus non-invading spheroid) in microfuge tubes and centrifuge at 4,000 rpm for 5 min at 4°C to separate the cells from the Matrigel.
  5.12. Carefully remove the supernatant and wash the pellet once with FACS buffer and centrifuge to remove any remaining Matrigel.
  5.13. Resuspend the pellet in 50 µl Hoechst 33342 (5 µg/ml in FACS buffer) and stain for 30 min at 37°C, protected from light.
  5.14. Wash cells by adding 1 ml FACS buffer and centrifuge at 400 *g* for 5 min.
  5.15. Resuspend cells in 300 µl FACS buffer and analyse by flow cytometry. Our procedure is outlined below and shown in **Figure S2**:
    5.1.1. Multicolor flow cytometry was performed on a BD Fortessa LSR SOPR. Cytometry set up and tracking beads (Cell Signaling Technology) was performed prior to acquisition after which the entire sample (at least 30,000 cells) was acquired under low sample speed.
    5.2.2. Compensation was performed as needed.
    5.3.3. Data analysis was performed using FlowJo with gating strategies as outlined (**Fig. S2**).
**CAUTION:** (i) As Matrigel liquefies at 4°C and solidifies at 37°C, it should be thawed on ice for at least 6 h before use. (ii) Pre-cool the 24-well glass bottom dish and P200/1000 pipette tips in the freezer to ensure even spreading of Matrigel. (iii) Ensure the entire surface of the glass bottom dish is covered with Matrigel and that no bubbles are generated.
**TIPS:** Although it is harder to ensure complete coverage of the plate using 50 µl Matrigel, this smaller volume improves confocal imaging quality.
**NOTES:** (i) Matrigel de-polymerizes after 7 d. (ii) CellTracker Red staining fades after 7 d so confocal images taken of mammosphere invasion into Matrigel should not be used for quantification by immunofluorescence. (iii) Phase contrast images of embedded spheroids should be taken daily from d 0 until the end of the assay at d7. Measurement of the invasive area of cells can then be measured by d 71397059140d 0. (iv) When using inhibitors, the desired concentration should be added to the Matrigel used to resuspend the mammospheres and to the BMM medium placed on top. Replenish BMM growth medium containing the inhibitors every 2 d. (v) unless fixed prior to flow cytometry analysis, transport cells on ice.

## ANTICIPATED RESULTS

To optimize Py8119 cell density for the generation of mammospheres, we prepared ultra-low attachment 6-well culture plates with 1 × 10^4^, 2.5 × 10^4^ or 5 × 10^4^ cells/well and observed the formation of aggregates for up to 30 d. We determined that 2.5 × 10^4^ cells was the most appropriate number to yield spherical mammospheres of approximately 500 µm diameter by 20 d of culture (**Fig. 2**). A higher density of cells produced irregular spheroids while a lower density of cells produced much smaller spheroids that were harder to handle and more likely to be lost during handling (data not shown). Previously, mathematical analysis has shown that solid acini above 500 µm in diameter display nutrient-starved and necrotic cells within a hypoxic core [3] and therefore our Py8119 mammospheres mimic the *in vivo* microenvironment of tumor growth.

**Figure 3:**
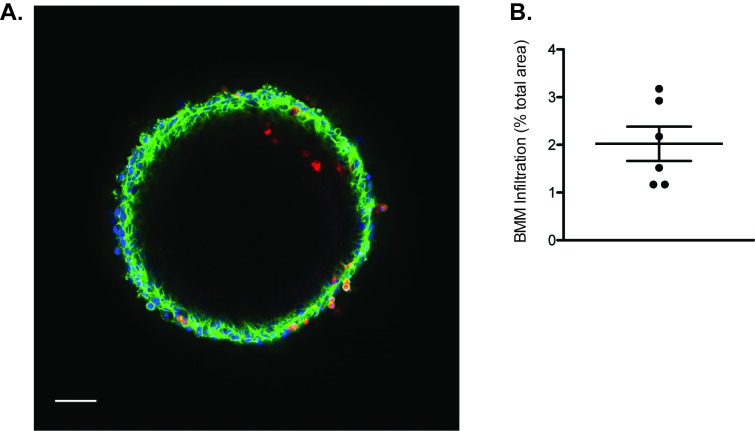
**BMMs infiltrate Py8119 mammospheres**. **A**. Py8119 mammospheres were infiltrated with BMM (red) for 3 d. After fixation and staining for F-actin (green), and DAPI (blue), mammospheres were analyzed by Confocal microscopy. Scale bar = 100 µm. **B**. Using > 3 mammospheres, the degree of infiltration relative to mammosphere area was quantified.

As mentioned previously, co-culture of tumor spheroids and macrophages permits separate examination of infiltration of macrophages into mammospheres and subsequent co-migration and invasion of tumor cells and macrophages into the surrounding matrix. First, we investigated the ability of mouse bone marrow-derived macrophages (BMM) to infiltrate into the mammospheres. After 3 d, most CellTracker Red labeled BMM are found within the outer several layers of tumor cells, however occasional BMM can be found deeper within the solid Py8119 spheroid (**Fig. 3**).

**Figure 4:**
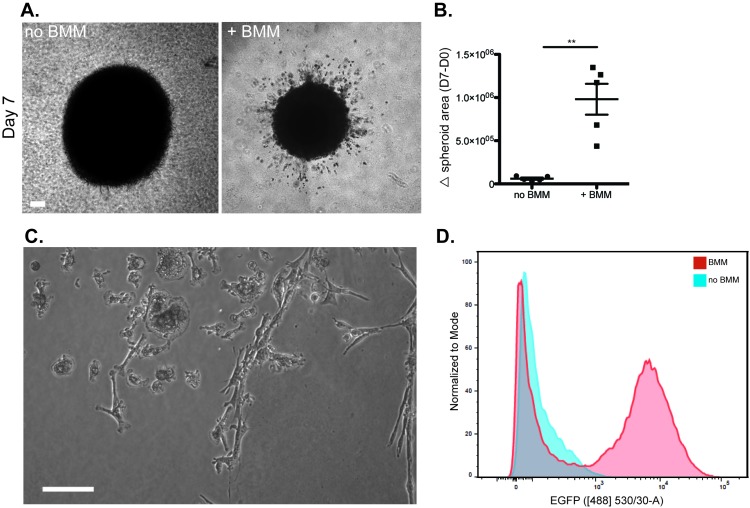
**BMMs promote tumor cell invasion into Matrigel**. **A**. Py8119 mammospheres, either infiltrated with BMM or not, were embedded into Matrigel and the area of invasive cells was measured after 7 d. Scale bar = 100 µm. **B**. Quantification of changes in mammosphere area, including surrounding invasive cells is shown, **P< 0.01. **C**. Phase contrast image (10x magnification) of a live culture BMM-infiltrated mammosphere embedded in Matrigel with streams of invading cells. Scale bar = 100 µm. **D**. Flow cytometric quantification of mammosphere invasion into Matrigel was carried out. Invasive EGFP^+^ Py8119 tumor cells were quantified by flow cytometry after gating for single cells and, in the absence of BMM, only EGFP^-^ was seen (first peak). In contrast, large numbers of EGFP^+^ tumor cells were seen invading the Matrigel from BMM-infiltrated mammospheres (second peak).

We next examined the ability of infiltrated BMM to promote tumor cell invasion into the surrounding matrix. Importantly, Py8119 spheroids, when embedded into Matrigel in the absence of co-cultured BMM, fail to become invasive (**Fig. 4A**). If BMM are allowed to infiltrate the spheroids prior to embedding in Matrigel, invasive activity of Py8119 cells is activated and they can be seen to co-migrate with BMM away from the spheroids and into the Matrigel (**Fig. 4A**). Rapid quantification of the degree of invasion can be carried out by subtracting the starting diameter of the spheroid from the final diameter of the invasive area after the cells have dispersed widely into the matrix (**Fig. 4B**). Higher power phase contrast imaging suggests that the linear streams of cells leaving the mammosphere consists of both BMM and tumor cells (**Fig. 4C**), and this was examined further using flow cytometry. After removal of the spheroid, flow cytometry of the depolymerized Matrigel was carried out to more closely examine the number of invasive tumor cells and BMM. The presence of BMM vastly increased the rate of invasion with a ratio of invasive tumor cells:invasive BMM of ~2:1 (**Fig. 4D**). Live imaging of invasion soon after embedding revealed the formation of tunnels dug by BMM through the Matrigel. The BMM could be seen shuttling backwards and forwards in these tunnels (**Fig. S1**, **Movie S1**).

Live imaging of Matrigel invasion by CellTracker Red-labeled BMM-infiltrated GFP-Py8119 mammospheres confirmed that invasive tumor cells were always accompanied by an associated macrophage (data not shown). Therefore, any single cells are macrophages.

**Figure 5:**
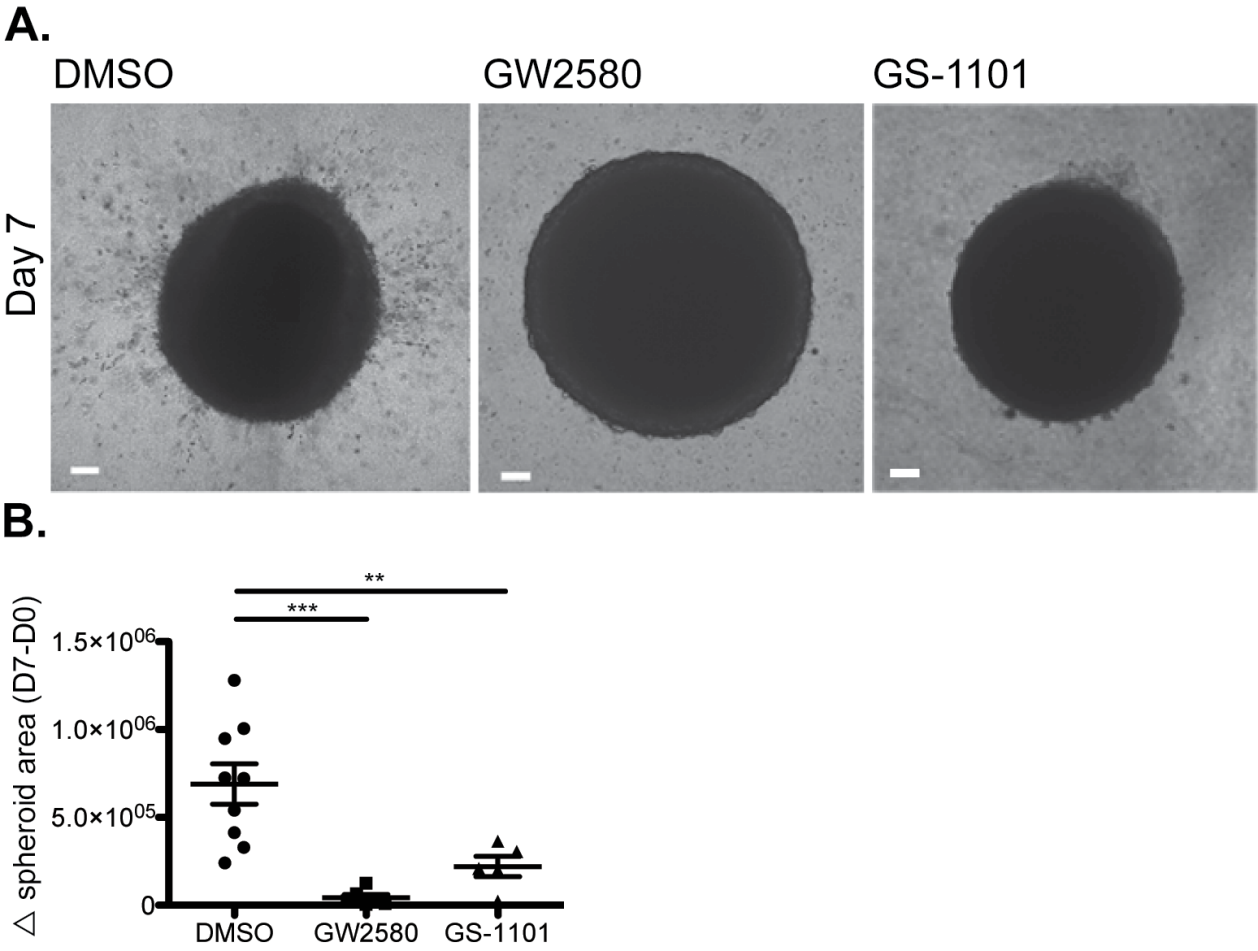
**Inhibition of BMM motility blocks tumor cell invasion**. **A**. Representative images of macrophage-infiltrated Py8119 mammospheres embedded in Matrigel in the presence of DMSO, CSF-1R inhibitor (GW2580; 2 µM) or PI3K p110δ inhibitor (GS-1101; 2 µM). After 7 d, the area of invasive cells was quantified for at least 3 mammospheres per condition. Scale bar = 100 µm. **B**. Quantification of tumor cell invasion. ***P< 0.001, **P< 0.01.

To test our hypothesis that BMM motility regulates tumor cell invasion, we compared the effects of an inhibitor of PI3K p110δ, GS-1101, that blocks macrophage motility [14] with a CSF-1R inhibitor, GW2580, in our mammosphere co-culture model. Consistent with our hypothesis, BMM-infiltrated mammospheres embedded in Matrigel in the presence of either GS-1101 or GW2580 failed to invade the surrounding matrix (**Fig. 5**). Since PI3K p110δ is a critical mediator of CSF-1-induced macrophage motility, signaling downstream of the CSF-1R is therefore integral to the promotion of tumor cell invasion by BMM. Importantly, although GW2580 blocks all CSF-1R signaling, this treatment did not deplete infiltrating macrophages from the mammosphere co-cultures (data not shown). Furthermore, as PI3K p110 is only expressed in hematopoietic cells such as macrophages, and not tumor cells, GS-1101 specifically targets macrophages in this context [14].

In summary, this robust co-culture system can be used to analyze BMM promotion of tumor cell invasion and screening for druggable targets. It is a useful tool to further dissect the mechanisms of promotion of tumor metastasis by macrophages.

## TROUBLESHOOTING

Tips for troubleshooting can be found in **Table 1**.

**Table 1:** Troubleshooting.

Step	Problem	Possible reason	Solution
2.4	Mammospheres do not form spherical shapes or take >30 d to form	• Cell density is too high	• Optimize cell number
		• Cell density is too low	• Manually swirl plate after 14 d in culture to encourage aggregation
		• Py8119 cells were allowed to get too dense in 2D culture	• Replenish growth medium with 2 ml every 4 d
			• Don’t allow Py8119 2D culture to become confluent prior to establishing 3D cultures
2.5	Mammosphere structure is disrupted during transfer	• Pipette tip is too narrow	• Use a wide bore pipette tip or cut the end off a tip with a sharp, sterile scalpel
		• Mammosphere was not transferred carefully	• Pipette mammosphere slowly and evenly
			• Allow mammosphere to sink to the bottom of the tip before ejecting
4.7	No/very little macrophage infiltration	• CellTracker Red staining is too faint	• If staining a very dense plate of macrophages, leave CTR solution on the cells for 35–40 min
		• Number of macrophages used to infiltrate is too few	• Increase concentration of CTR (**NOTE:** (i) Stock CTR is made up in DMSO, which is toxic to the cells at high concentrations. (ii) If a higher concentration of CTR is used, ensure the cells are allowed to recover for 3–4 h)
			• Increase macrophage number
			• Avoid scraping BMM too hard when lifting in EDTA
5.2	Matrigel layer contains air bubbles	• Pipette tip contained air while pipetting	• Ensure pipette tips are pre-cooled at −20°C prior to use
		• Matrigel was not thawed completely	• Use tips with a wider diameter or cut the tip with a sterile scalpel
			• Allow Matrigel to thaw on ice at 4ºC for at least 6 h
5.2	Matrigel is not evenly distributed in wells	• Matrigel was not added slowly and evenly to wells	• Ensure glass-bottom 24-well plate is pre-cooled at −20ºC prior to use and perform all steps on ice
		• Matrigel was not thawed completely	• Mix Matrigel by swirling the tube before each pipetting step and pipette the solution slowly and evenly into the centre of the wells
			• Swirl the plate to ensure even distribution of Matrigel
			• Allow Matrigel to thaw on ice at 4ºC for at least 6 h
5.3	Matrigel is not polymerizing	• Concentration of Matrigel is too low	• Use non-GFR Matrigel (high concentration) diluted to ≥ 8 mg/ml
5.5	Matrigel appears collapsed or retracted	• Medium was added too fast or with too much pressure	• Make sure medium is added very carefully
		• Plate was agitated	• Don’t remove the plate from the incubator for more than 5 min (except where necessary)
			• Transport and handle the plate carefully
5.7	Cells are not invading the surrounding Matrigel	• Macrophage cell number is too low	• Increase number of infiltrating macrophages
		• Matrigel concentration is too low	• Use non-GFR Matrigel with a concentration ≥ 8 mg/ml
		• Growth factor reduced (GFR) Matrigel is used	• Replenish media every 2 d

## Abbreviations used

BMM: bone marrow-derived macrophage
CSF-1: colony stimulating factor-1
CSF-1R: colony stimulating factor-1 receptor
EGFR: epidermal growth factor receptor
TAM: tumor-associated macrophage

## Supplemental Information

**Figure S1.** BMM-infiltrated Py8119 mammospheres were embedded in Matrigel for 3 d then subjected to live imaging by confocal microscopy for another 3 d (Nikon A1Si Confocal Microscope, Apo Plan 10x objective, NA 0.45, 6.85µm z-steps every 30 min).

**Figure S2.** Cells were gated in FSC-Area versus SSC-Area, after which FSA-Area versus FSC-Height was used to identify single cells.

**Movie 1**. BMM-infiltrated mammospheres were embedded in Matrigel for 3 d then imaged live by confocal microscopy for another 3 d (Nikon A1Si Confocal Microscope, Apo Plan 10x objective, NA 0.45, 6.85 µm z-steps every 30 min).
